# Promoting vitality in a sheltered workplace: a realist evaluation protocol

**DOI:** 10.1136/bmjopen-2025-107341

**Published:** 2025-10-27

**Authors:** Sanne Bom, Malin Hollaar, Semiha Denktaş, Paul Kocken

**Affiliations:** 1 Erasmus School of Social and Behavioural Sciences, Erasmus University Rotterdam, Rotterdam, The Netherlands

**Keywords:** QUALITATIVE RESEARCH, Workplace, Vulnerable Populations, PUBLIC HEALTH, Methods, Health Literacy

## Abstract

**Abstract:**

**Introduction:**

Employees in sheltered workplaces face greater challenges in maintaining a healthy and vitality-enhancing lifestyle due to physical, mental and psychosocial disabilities. While workplace health and vitality programmes are particularly relevant for this group, evaluation of programmes in this setting remains limited. This paper presents a protocol for a realist evaluation of a sheltered workplace’s vitality programme aimed at physical, mental and social sources of vitality.

**Methods and analysis:**

Using a multimethod design with adaptive qualitative methods, we developed an initial programme theory that describes how context and mechanisms of the sheltered workplace’s vitality programme influence the outcomes related to physical activity, healthy eating and social interaction. The protocol explains how we will collect and synthesise qualitative data, interviews, observations and focus group discussions, to refine and validate this initial programme theory and further evaluate the vitality programme. We aim to conduct at least 15 interviews, 15 observations, 3 focus group discussions and 1 final validating focus group. The study includes recipients of the intervention, namely individuals with varying work abilities, as well as other key stakeholders involved in the vitality programme. In this way, we will get insights into how to promote vitality-enhancing behaviours. Our context-sensitive methodology offers both scientific and practical value for future research in similar settings.

**Ethics and dissemination:**

Ethical approval was granted through the Ethics Review Committee of Erasmus University Rotterdam (application number: ETH2324-0939). Findings will be disseminated through presentations, conferences, social media and peer-reviewed publications.

**STRENGTHS AND LIMITATIONS OF THIS STUDY**

- The planned realist evaluation employs a multimethod qualitative design, including interviews, focus groups and observations, to ensure a multifaceted understanding of the context and mechanisms of a vitality programme.
- The use of multiple methods allowed us to include the perspectives of end-users, intervention designers and intermediates, thereby including an under-represented group of sheltered workplace employees.
- While the realist evaluation enables insights into the impact of the vitality programme, the exclusive use of qualitative methods limits the ability to draw conclusions in terms of cost-benefit and effectiveness of the vitality programme.

## Introduction

 While every individual has a unique set of skills and talents, some people require additional support to participate in the workforce due to physical, mental or psychological disabilities that limit their ability to work under standard conditions. Sheltered workplaces,^1^ also referred to as social firms^2^ or sheltered workshops,^3^ address these needs by offering adapted work environments. These settings offer job guidance and tailored support, enabling employees to make meaningful contributions to the labour market based on their capabilities.^1 3^ Despite this positive approach, sheltered workplaces record higher absenteeism and workers face more health-related challenges compared with employees of regular workplaces.^1^ Additionally, many sheltered workplace’ employees earn minimum wage or less, placing them in a lower socioeconomic position, a known risk factor for adverse health outcomes such as cardiovascular disease and diabetes.^4^ Given that adults spend a significant amount of time at work, the workplace is recognised as an important setting for promoting health and vitality.^5 6^

Vitality at work, as a combination of energy, motivation and resilience,^7^ is associated with employees’ perceived effective functioning and sustainable employability.^8^ Strijk *et al*^7^ propose that vitality is supported by modifiable sources of vitality at mental, physical and social levels. Mental vitality sources include, for example, sense of coherence; physical sources comprise lifestyle factors such as physical activity; and social vitality sources refer to the quality and quantity of social networks.^7^ Previous studies in the context of sheltered employment have explored factors related to employability, such as training needs and competence development,^9^ or the relation between levels of support, low autonomy and work engagement.^10^ However, few studies have focused on enhancing health and vitality or the implementation of workplace health promotion in the sheltered workplace setting.^1 2^ Van Heijster *et al*,^1^ in their research about participation in workplace health promotion programmes, suggest that participation can be promoted by encouraging employees to work together, tailoring activities and connecting activities with employees’ daily lives. Kordsmeyer *et al*,^2^ in their study of social firms, identified needs and challenges in the implementation of workplace health promotion in sheltered employment. The current study seeks to close a research gap by examining not only challenges to implementation but also effective elements that contribute to the outcomes of a vitality-enhancing programme.

We will conduct a realist evaluation to unravel the context, mechanisms and outcomes of a vitality-enhancing workplace programme (hereafter referred to as the vitality programme) of a sheltered workplace in Rotterdam (<2000 employees). The goal of the vitality programme that is being studied is to enhance employees’ long-term employability, through impacting physical activity, healthy eating and social interactions. Although the vitality programme’s content is predesigned, its activities are adapted to the capabilities of the diverse group of employees (eg, employees with physical impairments walk shorter rounds than employees who are better off physically). This tailoring to the needs of sheltered workplace employees ensures a better alignment. However, it also makes evaluating the programme’s outcomes more complex, highlighting the need for an appropriate evaluation method that can capture contextual variation.

The realist evaluation protocol supports a step-by-step exploration of the full picture of the components of a vitality programme in a sheltered workplace. We aim to develop a framework to help understand how the programme is organised in terms of context, mechanisms and outcomes. We will examine contextual elements such as the workplace setting and employees’ capabilities, mechanisms like tailoring the programme to employees’ needs, and how these mechanisms impact outcomes related to vitality-enhancing behaviours such as physical activity, healthy eating and social interactions. Our research will answer the following question: *How do contextual elements and mechanisms of a vitality programme in a sheltered workplace contribute to the intended outcomes?*

## Intervention design

The vitality programme is implemented at a sheltered workplace in Rotterdam, The Netherlands, which provides sheltered employment to approximately 2500 employees across various locations in the city. There is a large diversity in employees and the reasons why they require sheltered employment, ranging from intellectual, mental or physical disabilities to socioeconomic or psychosocial challenges that complicate regular employment. The vitality programme is an effort to reduce absenteeism and improve worker’s vitality, health and job satisfaction. It exists as a combination of activities contributing to the three dimensions of vitality (resilience, motivation and energy) by addressing physical, mental and social sources of vitality.^7^

Most activities are aimed at physical sources of vitality, relating to healthy eating and physical activity. Regarding healthy eating, free fruit is supplied by an external provider on a daily basis. Boxes filled with fruit and vegetables are placed in the different departments and locations of the sheltered workplace. Some departments actively distribute the fruit, with or without the help of an employee, while other departments simply place the box in a public space without instructions. Moreover, the canteens offer a selection of healthy food options next to the regular offer of snacks. Physical activity is promoted through weekly scheduled walks during working hours, daily workplace walks as energisers between tasks and encouraging employees to move through fitness machines (ie, treadmill, bike) placed in the departments. Adaptations to the workplace such as putting storage further away are made to stimulate physical activity during work activities. Besides physical sources of vitality, various individuals (ie, vitality coordinator, team coaches) take initiative to organise activities related to social and mental sources of vitality. These activities aim to increase job satisfaction, social connections between employees and work morale. For instance, a yearly ‘Fit Festival’ takes place, in which employees can connect socially while learning about topics such as nutrition, physical activity, smoking cessation and financial well-being. Next to that, more spontaneous activities such as a yearly bingo or table tennis tournaments are organised on a department level.

The programme is coordinated by a ‘vitality coordinator’ and a management team responsible for sustainable employability. The management team and vitality coordinator decide more formally on the offer of the vitality programme, but there are no set guidelines on participation nor frequency of use. In practice, the programme is often adapted to specific needs of employees, in which their direct supervisors, that is, team coaches, play a role. Adapting includes using understandable language or matching activities to the daily work activities of employees. Activities are offered throughout the year and adapted accordingly (eg, longer walks in summer than in winter).

## Methods

We used realist evaluation, a theory-driven evaluation method to evaluate the vitality programme.^11^,^13^ A realist evaluation involves the development of a programme theory, which consists of context-mechanism-outcome (CMO) configurations that capture a representation of reality. These configurations describe how a mechanism (M), triggered in a certain context (C), results in a certain outcome (O).^13^ Literature poses slight differences in the definitions of what CMOs entail.^14 15^ In this protocol article, we follow Mukumbang *et al*^14^ and Smeets *et al*^15^ definition where M refers to underlying determinants of social behaviour generated in certain contexts, C are the conditions that enable or constrain the activation of programme mechanisms, and O are the (intended) effects of programme activities.^14 15^ For example, when evaluating a patient dietary health intervention, the C may involve the capabilities of healthcare staff to motivate patients, an M could be tailoring the intervention to patients to match their preferences and the intended O would be healthier eating behaviours such as increased fruit intake. A realist evaluation does not prescribe a predefined combination of research methods.^12^ The current evaluation of the vitality programme employs a multimethod design^16^ consisting of various qualitative methods. Our realist evaluation of the vitality programme consists of four phases outlined in this protocol. In this research project, we are following the RAMESES-II reporting standards for realist evaluations.^13^ The different phases of our realist evaluation are depicted in table 1. In the first phase, we developed an initial programme theory, which served as input for the subsequent phases of our realist evaluation (see table 1). Phase 2 is for data collection for theory refinement (a realist term for adapting and improving the initial theories), phase 3 is for analysing data and further theory refinement and in phase 4, we will perform a final validation of the programme theory and evaluative synthesis. The study commenced in July 2024 and is expected to continue until October 2025. The next sections provide elaborate descriptions of our realist evaluation phases and planned methods.

**Table 1:** Phases of our realist evaluation and related methods

Phase in realist evaluation	Methods
Phase 1: development of the initial programme theory (completed in January 2025)	Stakeholder interviews, logbook data, desk research of available documentation
Phase 2: data collection for theory refinement	Focus group discussions with team coaches, interviews with employees, observations at the sheltered workplace
Phase 3: data analysis and theory refinement	Data synthesis matrix
Phase 4: final validation of the programme theory and evaluative synthesis	Focus group discussion to validate the refined programme theory

### Phase 1: development of the initial programme theory

**Figure 1:**
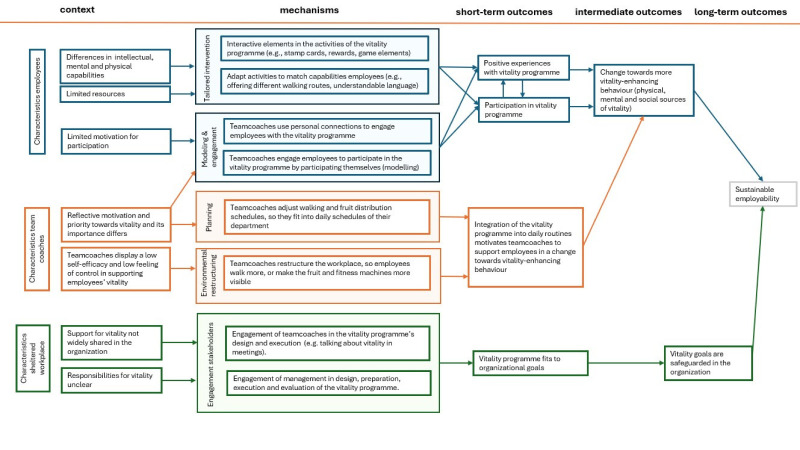
Initial programme theory displaying context, mechanisms and short-term and long-term outcomes of the vitality programme.

The first phase of a realist evaluation (RE) typically involves developing an initial programme theory to explore how different mechanisms generate specific outcomes across various contexts.^17 18^ To develop initial programme theory and create a comprehensive understanding of the vitality programme, we used a combination of methods, that is, desk research of available documentation (attending stakeholders’ meetings and describing these in a logbook) and 10 semistructured interviews with managers and other stakeholders involved in the vitality interventions. This number of interviews was deemed sufficient to reach data saturation, considering the small number of stakeholders involved in vitality interventions. From these data, we identified contextual factors that impact mechanisms and relevant outcomes. These were first presented in CMO configurations in an initial programme table (see table 2) and thereafter through iterative discussions refined into an initial programme theory, presented in figure 1. To guide the development of this initial programme theory, we used constructs of Damschroder’s Consolidated Framework for Implementation Research^19 20^ and the Behaviour Change Wheel.^21^ The next paragraph will shortly introduce the initial programme theory (figure 1).

**Table 2:** Initial programme table: context-mechanism-outcome (CMO) configurations of the initial programme theory

	CMO configurations: employees
1a	Employees have different intellectual, mental and physical capabilities (c), which is why the vitality programme is adapted to employees’ abilities (eg, simple language, adapted walking routes) (m), ensuring employees can actively participate and have positive experiences with the vitality programme (o).
1b	Employees are not always motivated to participate in the vitality programme (c), which is why the interventions are designed to be accessible and interactive with (game-based) formats (m), leading to a positive experience with the interventions (o).
1c	Because employees are not always motivated to take part in vitality-related interventions (c), team coaches actively engage employees to participate using the personal connections with employees (m), which increases participation in the interventions (o).
1d	Because employees are not always motivated to take part in vitality-related interventions (c), team coaches actively encourage and motivate employees to participate by providing an example of participating themselves (m), which increases participation in the interventions (o) and a positive experience with the interventions (o).
1e	Employees have limited resources (c), which is why the vitality programme offers free activities adapted to working hours using rewarding elements (m), increasing participation in the programme (o).

	CMO configuration: team coaches
2a	Team coaches vary in motivation to enhance employees’ vitality-related behaviours (c), but those who do fit activities of the vitality programme into daily schedules of their department (m) are motivated to support employees in a behavioural change towards vitality-enhancing behaviour (o).
2b	Not all team coaches feel like they are able to support employees’ vitality due to factors as workload or priority for vitality (c), that is why the vitality programme is integrated into daily tasks and routines (m). Because of this, team coaches are motivated to support employees in a behavioural change towards vitality-enhancing behaviour (o).
2c	Team coaches vary in motivation to enhance employees’ vitality-related behaviours and do not always feel like they are in control of their employees’ vitality (c), but through environmental restructuring (m), they stimulate vitality-enhanced behaviour of employees (o).

	CMO configuration: organisational level
3a	Roles and responsibilities regarding vitality are not always clearly assigned (c), but by engaging team coaches in the vitality programme’s design and execution (m), the vitality programme fits better to the organisational goals (o).
3b	There is no defined organisation-wide support for vitality-enhancing programmes (c), but by engaging management members in design, preparation, execution and evaluation of the vitality programme, the programme fits better to organisational goals (o), which helps build broader support for vitality (o).

c, context; m, mechanisms; o, outcomes.

Figure 1 depicts an abstract version of our initial programme theory, while the following paragraph offers a more detailed explanation. We identified three levels of stakeholders involved in the vitality programme: employees as recipients, team coaches in an intermediary role and the organisation as the facilitator.

The first row of figure 1 (blue boxes) presents the CMO configurations relating to the employees. Our findings indicate that employees differ in their capabilities due to underlying factors such as long-term physical, mental or social challenges, as well as limited resources (ie, money). These factors may influence programme outcomes like participation and positive experiences, and eventually a change towards vitality-enhancing behaviour on physical, mental and social levels. For example, limited resources may restrict employees’ ability to purchase healthy food, potentially increasing their motivation to participate in free activities such as fruit provision at work. Next to these contextual elements, we found mechanisms that appear to influence outcomes on an employee level: tailoring of interventions, social connections between colleagues and role modelling. These mechanisms are often put in place by team coaches, but directly influence employee participation. Tailoring involves adapting programme activities to suit employees’ capabilities, for instance through the use of simple language and visuals instead of long texts to aid comprehension. It may also include the use of interactive elements, such as a walking app that rewards employees with coupons after a certain amount of steps. Next to tailoring, team coaches use role modelling to engage employees. For instance, by showing the ‘right’ behaviour by participating in activities themselves. Moreover, they encourage participation by adopting a personalised approach, such as addressing employees by name or remembering their preferences. We also found that social connections between colleagues seem to be a mechanism to increase participation. Combining social and vitality-enhancing activities, for instance during a shared fruit break, seems to encourage employees to eat healthier. Through these mechanisms, employees are more likely to participate and have positive experiences with the vitality programme, which is expected to contribute to a behavioural change towards vitality-enhancing behaviour such as eating more healthier food, more physical activity and increased social interaction.

The second row (orange boxes) focuses on CMOs related to team coaches and their role in adjusting the vitality programme to daily activities. We found that team coaches have different priorities and motivation for vitality and vitality-related actions in the programme. While some team coaches are motivated to engage in vitality-enhancing activities for employees, other team coaches feel less of a capacity to influence employees’ vitality, which we referred to as low self-efficacy. However, team coaches do seem to engage in planning of vitality-enhancing activities by adjusting fruit distribution moments to suit the department, or by allowing time for workplace walking activities. Moreover, we found that team coaches restructure the workplace to make sure that employees move more during their work activities. By incorporating activities of the programme into daily tasks and routines, team coaches are supporting employees in a change towards vitality-enhancing behaviour.

The third row (green boxes) shows CMOs on an organisation level, illustrating the involvement and engagement of a management team in organising and safeguarding the vitality programme. Our findings reveal variations in how employees at a managerial level prioritise vitality. For example, while some higher-level employees express that they consider the vitality programme important, they also express an inability to act due to limitations in their role description. A mechanism we found that could increase support for the programme is engaging both team coaches and management team members in the vitality programme’s design, execution and evaluation. Eventually, this could increase a better fit with the organisational goals and managerial employees within the sheltered workplace. Structural safeguarding of vitality-enhancing actions in organisational goals could improve sustainable employability in the long run.

Structural factors such as organisational policy, workplace health and safety regulations and the availability of budget for preventive measures potentially influence the effectiveness and long-term sustainability of the vitality programme. For instance, differences in employment contracts impact employees’ salary levels, benefits and levels of autonomy. These disparities may affect not only participation in the vitality programme but also perceptions of fairness and long-term engagement at work. The vitality programme takes place in the context of policy, finances, contract forms or power relations within the organisation, which will not be systematically mapped but will be taken into account as explanatory contextual factors when relevant. Their influence is recognised and will be taken into account when relevant in the next phases of the research and further refinement of the programme theory.

The CMO configurations pictured in the initial programme table (table 2) and the initial programme theory (figure 1) serve as a starting point for the data collection in the next phases, which will eventually result in a more detailed and refined programme theory.

### Phase 2: data collection for theory refinement

The second step in a realist evaluation is the empirical process of systematically refining and testing the initial programme theory.^17^ We will collect qualitative data on different participant levels. Our multimethod approach will include interviews with employees about the vitality programme, observations at the sheltered workplace, focus group discussions with team coaches and logbook data. Table 3 provides an overview of how we plan to investigate each CMO configuration using at least one of the abovementioned data sources. This structured approach ensures that all configurations will be addressed through planned data collection and analyses. With our approach, we will assess the short-term outcomes, such as experiences and participation, and some intermediate outcomes, such as integration in daily routines, of the vitality programme (see figure 1), as well as the impact of the programme components and contextual factors. However, longer-term goals, such as a sustainable employability, are beyond the scope of the current research.

**Table 3:** Context-mechanism-outcome (CMO) configurations, link to data collection methods and purpose in realist evaluation

Method	CMO configuration	Sample size/frequency	Purpose in realist evaluation
Focus groups with team coaches	1c, 1d, 1e, 2a, 2b, 2c, 3a	n=15 team coaches, 3 focus groups at the three main locations of the sheltered workplace	Understanding team coaches’ perspectives on CMO relationships
Interviews with employees, semistructured	1a, 1b, 1d, 1e	n=15–20 employees	Exploring firsthand experiences of short-term outcomes and factors influencing these changes
Observation workplace	1a, 1b, 1c, 1d, 1e, 2a, 2b, 2c, 3a	n=10–15 observations	Insights into the behaviour of employees and how vitality and corresponding behaviours are displayed within the workplace
Logbook	1b, 3b	–	Providing context for interpreting the findings and alignment with programme theory (PT), supporting data triangulation
Validation of PT through stakeholder focus group discussion	3a, 3b	1 focus group with n=8 stakeholders	Validation of our PT with stakeholders involved with the vitality programme

#### Focus group team coaches

The aim of three semistructured focus group discussions is to gain more insight into the process of delivering the vitality programme. The discussions will include questions on team coaches’ perceptions of employee’s vitality-related behaviour, barriers and facilitators to employee participation in the vitality programme and team coaches’ perceived role in motivating and engaging employees. On a more organisational level, the discussion will elaborate on the role of team coaches in promoting vitality-enhancing activities within the organisation, processes for proposing new activities, needs and perceived obstacles (eg, workload, organisational priorities). We aim to conduct three focus group discussions, one at each of the main sites of the sheltered workplace, including at least five team coaches each time, to ensure coverage of team coaches’ perspectives across organisational contexts and to provide sufficient depth and diversity of insights.

#### Employee interviews

The semistructured interviews will explore employees’ experiences with the activities of the vitality programme and assess how it operates in practice. Specifically, we will examine how the activities are perceived (ie, in terms of interactivity, relevance, enjoyment), whether they align employees’ needs and capabilities, and if employees changed their behaviour. Taking into account the diversity in physical, mental and social capabilities within the employee population, semistructured interviews have been chosen as the most suitable method because they allow researchers to adapt questions based on participants’ level of understanding. To enhance inclusivity, we will adapt the interviews to increase comprehensibility by using clear language, adjusting the length of the interview to a maximum of 10 min and prioritising a higher quantity of total interviews over lengthy in-depth interviews. Researchers will train their interview skills specified to this population beforehand. Next to possible comprehension issues, the willingness to participate in an interview might differ between employees. Employees might be sceptical about being interviewed by our team of external researchers. To reduce any possible distrust, we will ask team coaches to act as intermediates by facilitating contact and introducing the research. Informed consent will be explained verbally and obtained both verbally and in writing. These adaptations will ensure that our methods are accessible and appropriate for the employees. We aim to conduct 15–20 semistructured interviews with employees across different locations during working hours which is an empirically supported estimate for achieving saturation in qualitative research considering our specific research objectives.^22^

#### Observation sheltered workplace

Through workplace observations, we want to obtain insights about the everyday context of the sheltered workplace employees, focussing particularly on how various sources of vitality (eg, physical, mental, social)^7^ are displayed within the workplace. Observations will follow a predetermined semistructured format based on these vitality sources, with open sections to capture unanticipated insights.^23^ Regarding physical sources of vitality, the format will record data about who takes fruit, how often and how much is taken and whether fruit distribution is part of a daily routine. For physical activity, it will record use and duration of fitness machines, strategies to motivate employees and whether employees are given time and space to use the equipment. Participation in workplace walking activities will also be tracked. Mental and social sources of vitality will be recorded, including workplace activities that happen on a department level, such as playing games, dance breaks or ping pong tournaments. The format will have an open section to record any other vitality-enhancing behaviours and contextual factors relevant to the outcomes of the vitality programme.

Researchers will conduct passive observations without interfering in daily tasks of employees. Observations will take place during working hours, covering both morning and afternoon shifts on varying weekdays, to account for differences between the days. Consent for observations will be obtained beforehand by team coaches, who will inform the employees about the researcher’s presence. We aim to do at least 15 observations across the three main locations and on different departments until data saturation^23^ is reached.

#### Logbook

To document the study process and decisions made throughout, researchers will keep notes in a logbook. This contains notes of meetings with the sheltered workplace’ stakeholders that are involved in this evaluation project and the vitality programme. It captures key decisions made during the study and intervention process, feedback received by stakeholders and personal reflections. As such, the logbook will provide context for interpreting the findings and alignment with programme theory, supporting eventual data triangulation in phase 3 of the research.

### Phase 3: data analysis and theory refinement

In phase 3, we will analyse each type of collected data separately, followed by an integrative synthesis to triangulate findings and refine the initial programme theory. Refinement, in this realist context, refers to adapting the theory based on emerging insights. Audio recordings (focus groups and interviews from phase 2) will be verbatim transcribed, and written observations digitalised. Each data source will then be analysed separately using Braun and Clarke’s^24^ six-step thematic analysis in ATLAS.ti (V>24), applying both deductive and inductive coding. Deductive coding will be guided by the CMO configurations from the initial programme theory, while inductive coding will allow for the emergence of new themes and mechanisms to inform theory refinement.

After analysis and coding, we will develop a synthesis matrix to integrate findings from all data sources to the initial CMO configurations. This matrix will enable systematic testing of the CMO configurations by identifying which data support or refute them. Where findings across data sources align, existing CMO configurations of the initial programme theory will be considered supported. In case of divergence, inconsistencies will be analysed to explore variations in CMOs. If necessary, CMO configurations will be revised or new CMO configurations will be developed.

Insights from the integrated data in the synthesis matrix, combined with logbook data, will guide an iterative refinement of the programme theory. This process will be guided by collaborative discussions with the research team. Researcher decisions during triangulation will be documented in the logbook to increase transparency.

### Phase 4: final validation of the programme theory and evaluative synthesis

In the final phase of our realist evaluation, the refined programme theory in phase 3 will be validated through a focus group with stakeholders from the sheltered workplace. Feedback and insights from this session will inform a final round of adjustments to the programme theory. Once finalised, through evaluative synthesis, the validated programme theory will be used as a framework to answer our research question: *How do contextual elements, and mechanisms, of a vitality programme in a sheltered workplace contribute to the intended outcomes?*

### Patient and public involvement

Patients were not involved in this protocol, as the sheltered workplace cannot be considered a clinical setting. However, input collected during meetings with the sheltered workplace contact persons was incorporated in the design of our study. Final findings will be shared with the sheltered workplace employees using accessible, low-literacy communication methods (eg, through a work newspaper). In addition, results will be disseminated to other sheltered workplaces through presentations.

## Discussion

Although research on vitality and workplace health promotion has gained popularity, few have focused on how such interventions function within the unique and layered context of a sheltered workplace.^25 26^ Preliminary results from our initial programme uncover the context and mechanisms of a vitality-enhancing workplace programme. This protocol displays our planned methods for refinement and adaptation of our initial programme theory.

The realist evaluation approach is a good fit for our study’s context and target group due to its flexibility and adaptability. Realist evaluations embrace the complexity of daily lives and settings.^27^,^29^ Whereas traditional methods often focus on outcomes and assume linear causality, realist evaluations allow to unpack the mechanisms and contextual factors by which these interventions may or may not lead to the intended outcomes, in our case a change in vitality-enhancing behaviours.^17 28^ This approach enables us to generate insights that are not only scientifically robust but also practically relevant for tailoring interventions to real-life conditions for the target group.^29 30^

Methodologically, our protocol demonstrates ways in which research can be realistically and ethically adapted to include underrepresented groups. Previous research has recognised the importance of including under-represented groups in research, but encounters barriers such as logistical challenges, trust issues, assumptions about participants’ abilities that hinder their inclusion and issues regarding the provision of informed consent to participate.^31^,^33^ Given the variety of reasons individuals might enter sheltered employment, we anticipated various challenges in including this target group in our research. By using qualitative and adaptive methods, designed in consultation with practitioners within the sheltered workplace itself and other researchers working with similar target groups, we hope to overcome these challenges. Rather than framing our target populations as ‘difficult’ or ‘hard-to-reach’, we adopt a strength-based lens focusing on possibilities rather than solely focusing on limitations and we take a systems approach, to stress that the responsibility for the vitality-related outcomes does not lie only with the individual employees. After all, behaviour is shaped by many factors, including social determinants of health such as working conditions, income, housing and educational opportunities all contributing to persistent health inequities.^34^ At the first stage of our evaluation in developing the CMO, little mention was made of structural factors of the social organisation of work while work stress and little control of one’s work are important determinants of health.^34^ These contextual factors will be taken into account when explaining mechanism and outcomes of the vitality programme. With this protocol, we aim to demonstrate how research methods can be adapted to better align with the context and needs of the target population, while still enabling the identification of key working elements through the involvement of a range of stakeholders. In doing so, we seek to overcome potential challenges related to participant engagement and communication.

The central concept in our study is vitality, which is conceptualised in current research as a multidimensional state that encompasses energy, resilience and motivation and with vitality sources on physical, mental and social levels.^7^ However, it remains a developing construct with ongoing debates about its precise definition and measurement.^35 36^ By exploring vitality-enhancing activities within the context of sheltered employment, we will offer practical insights into how the concept can be operationalised and applied for individuals in sheltered work environments. Additionally, while behaviour change theory offers valuable frameworks for understanding general human behaviour, effective interventions require careful tailoring to their specific contexts. This is crucial to avoid overlooking important nuances and situation-specific factors that actually drive behaviour.^37 38^ Therefore, to develop impactful behaviour change interventions within the setting of sheltered employment, we extend the applicability of behaviour change theory to accommodate workers with a variety of physical, mental and intellectual competences.

### Strengths and limitations

In designing this study, we chose a multimethod design consisting of multiple qualitative data sources, rather than employing a mixed-methods design with quantitative elements.^16 27^ We deemed a multimethod qualitative design to better align with the capabilities of our target group in comparison to quantitative methods such as questionnaires, less suitable for our target group with limited cognitive abilities. To strengthen the richness and validity of findings, we incorporated a diverse range of qualitative data sources, such as interviews, focus groups and employee observations, including the variety of parties at different levels, involved in a sheltered workplace. Together, we believe this ensures a multifaceted understanding of the contexts and mechanisms of the vitality programme.

A realist evaluation is suitable for in-depth, contextual insights into mechanisms and processes. However, due to this sensitivity to context, statements about the effectiveness of the vitality programme are not easily generalised to other settings. Although generalisability is not the primary goal of a realist evaluation, this remains a potential limitation. Additional quantitative research should be performed to provide a stronger evidence base and to improve generalisability to other workplace settings. Nonetheless, through triangulation of several qualitative data sources, we aim to provide transparent, actionable and transferable insights that are specific enough for practical use for our evaluation of a sheltered workplace, while also being broad enough to be applied beyond one single case. As such, our programme theory offers a framework that can be used by others to adapt and test in comparable settings.^29^

Another difficulty of applying the realist method is that it needs iterative, non-linear thinking. Since it does not have a fixed, step-by-step design, there might be a lack of methodological guidance.^29^ Realist evaluations evolve during data collection and analysis.^27^ However, we explicitly acknowledge this adaptive nature in our protocol, particularly when evaluating a dynamic, ongoing programme like ours.

## Ethics and dissemination

This study protocol has obtained formal ethical approval through the Ethics Review Committee of Erasmus University Rotterdam (application number: ETH2324-0939). The research is conducted by researchers from Healthy’R, a collaborative knowledge centre from Erasmus University Rotterdam and the municipality of Rotterdam, linking practice, policy and research. In addition to this protocol and a peer-reviewed article reporting the findings, a policy report will be prepared for internal use and dissemination to other sheltered workplaces and municipalities. Results will be shared through presentations, conferences, social media and the sheltered workplace’s local newspaper.

Upon project completion, original data including audio recordings, transcripts and observational notes will be securely stored in a restricted-access online environment accessible only to the research team. Metadata will be shared via the Open Science Framework after publication and completion of the investigators’ PhD. All published materials will ensure participants’ anonymity. Data will be retained for a period of 10 years.
