# Supplementary material for: Promoting vitality in a sheltered workplace: a realist evaluation protocol
Source: BMJ Open. 2025 Oct 27;15(10):e107341. doi: 10.1136/bmjopen-2025-107341 (PMC12570957; doi:10.1136/bmjopen-2025-107341)
Supplement: Supplementary file 1 [file bmjopen-2025-107341.reviewer_comments.pdf]

# PEER REVIEW HISTORY

BMJ Open publishes all reviews undertaken for accepted manuscripts. Reviewers are asked to complete a checklist review form and are provided with free text boxes to elaborate on their assessment. These free text comments are reproduced below.

## ARTICLE DETAILS

### Title (Provisional)

Promoting Vitality In A Sheltered Workplace: A Realist Evaluation Protocol

### Authors

Bom, Sanne; Hollaar, Malin; Kocken, Paul; Denktas, Semiha

---

## VERSION 1 - REVIEW

---

| | |
|---|---|
| **Reviewer** | **1** |
| **Name** | **Currie, Jane** |
| **Affiliation** | **Queensland University of Technology, School of Nursing** |
| **Date** | **30-Aug-2025** |
| **COI** | **None** |

---

Dear Authors,

Thank you for your submission, it is clearly written, and the methods of the proposed study are robust. My only feedback is to consider providing a brief explanation of what is meant by context, mechanism and outcome. From my own application of realist evaluation these three elements can be open to interpretation and it could be valuable to define what is meant by each and provide an example.

Best wishes

---

## VERSION 1 - AUTHOR RESPONSE

**Comment 7:** Thank you for your submission, it is clearly written, and the methods of the proposed study are robust. My only feedback is to consider providing a brief explanation of what is meant by context, mechanism and outcome. From my own application of realist evaluation these three elements can be open to interpretation, and it could be valuable to define what is meant by each and provide an example.

**Response 7:** Thank you for the compliments, we appreciate it. Indeed, context, mechanism and outcomes are described differently in the scientific literature. We agree that it is valuable to define what is meant by each and to provide an example. Therefore, on page 5, line 140 to 148 we added a new paragraph:

*"Literature poses slight differences in the definitions of what CMOs entail [14,15]. In this protocol article, we follow Mukumbang et al (2020) and Smeets et al. (2022) definition where a mechanism (M) refers to underlying determinants of social behaviour generated in certain contexts, context (C) are the conditions that enable or constrain the activation of program mechanisms, and outcomes (O) are the (intended) effects of program activities (Mukumbang et al., 2020; Smeets et al., 2022). For example, when evaluating a patient dietary health intervention, the context (C) may involve the capabilities of healthcare staff to motivate patients, a mechanism (M) could be tailoring the intervention to patients to match their preferences and an intended outcome (O) would be healthier eating behaviours such as increased fruit intake."*

We have added two additional references to clarify that differences in CMO definition exist, and described elsewhere:


Mukumbang, F. C., Marchal, B., Van Belle, S., & Van Wyk, B. (2020). Using the realist interview approach to maintain theoretical awareness in realist studies. *Qualitative Research*, *20*(4), 485-515. https://doi.org/10.1177/1468794119881985

Smeets, R. G. M., Hertroijs, D. F. L., Mukumbang, F. C., Kroese, M. E. A. L., Ruwaard, D., & Elissen, A. M. J. (2022). First Things First: How to Elicit the Initial Program Theory for a Realist Evaluation of Complex Integrated Care Programs. *The Milbank Quarterly*, *100*(1), 151-189. https://doi.org/10.1111/1468-0009.12543


Additionally, thank you for the reminder of BMJ Open referencing style. Accordingly, we have made some revisions in the referencing style changing it from APA referencing to Vancouver / BMJ Open guidelines for referencing.

---
